# Phenological Responses to ENSO in the Global Oceans

**DOI:** 10.1007/s10712-016-9391-1

**Published:** 2016-11-09

**Authors:** M.-F. Racault, S. Sathyendranath, N. Menon, T. Platt

**Affiliations:** 1 Plymouth Marine Laboratory (PML), Prospect Place, The Hoe, Plymouth, UK; 2 National Centre for Earth Observation (NCEO), PML, Plymouth, PL1 3DH UK; 3 ESA Living Planet Fellowship, PML, Plymouth, PL1 3DH UK; 4 INDO-European Research Facilities, Studies on MARine Ecosystem and CLIMate in India (INDO-MARECLIM), NERCI, Kochi, Kerala 682016 India; 5 Nansen Environmental Research Centre India (NERCI), 6A, Oxford Business Centre (6th Floor), Sreekandath Road, Ravipuram, Kochi, Kerala 682016 India

**Keywords:** ESA climate change initiative, Ocean colour, Phytoplankton, Phenology, El Niño–southern oscillation, Climate impact

## Abstract

Phenology relates to the study of timing of periodic events in the life cycle of plants or animals as influenced by environmental conditions and climatic forcing. Phenological metrics provide information essential to quantify variations in the life cycle of these organisms. The metrics also allow us to estimate the speed at which living organisms respond to environmental changes. At the surface of the oceans, microscopic plant cells, so-called phytoplankton, grow and sometimes form blooms, with concentrations reaching up to 100 million cells per litre and extending over many square kilometres. These blooms can have a huge collective impact on ocean colour, because they contain chlorophyll and other auxiliary pigments, making them visible from space. Phytoplankton populations have a high turnover rate and can respond within hours to days to environmental perturbations. This makes them ideal indicators to study the first-level biological response to environmental changes. In the Earth’s climate system, the El Niño–Southern Oscillation (ENSO) dominates large-scale inter-annual variations in environmental conditions. It serves as a natural experiment to study and understand how phytoplankton in the ocean (and hence the organisms at higher trophic levels) respond to climate variability. Here, the ENSO influence on phytoplankton is estimated through variations in chlorophyll concentration, primary production and timings of initiation, peak, termination and duration of the growing period. The phenological variabilities are used to characterise phytoplankton responses to changes in some physical variables: sea surface temperature, sea surface height and wind. It is reported that in oceanic regions experiencing high annual variations in the solar cycle, such as in high latitudes, the influence of ENSO may be readily measured using annual mean anomalies of physical variables. In contrast, in oceanic regions where ENSO modulates a climate system characterised by a seasonal reversal of the wind forcing, such as the monsoon system in the Indian Ocean, phenology-based mean anomalies of physical variables help refine evaluation of the mechanisms driving the biological responses and provide a more comprehensive understanding of the integrated processes.

## Introduction

Phytoplankton are microscopic unicellular algae living in the upper layer of oceans. Through chlorophyll and associated pigments that they contain, phytoplankton carry out photosynthesis, which contribute to the oceanic uptake of the CO_2_ emitted to the atmosphere every year. This CO_2_ sink is part of a very active, natural carbon cycle, through which phytoplankton in the surface layer of the ocean fix CO_2_ into organic matter, some of which subsequently sinks below the mixed layer. Through this process, phytoplankton help to modulate the increase in atmospheric CO_2_ that results from the burning of fossil fuels. Moreover, phytoplankton are at the base of the food chain and transfer energy to higher trophic levels. This transfer of energy has a knock-on effect on fisheries and dependent human societies especially in highly productive and coastal upwelling regions. Thus, phytoplankton are key players in the planetary carbon cycle and provide important services to the society.

Several metrics have been developed to quantify variations in phytoplankton populations. These metrics form ecological indicators, which provide systematic and objective information about the state of the marine ecosystem (Platt and Sathyendranath 2008). Analysis of a suite of indicators belonging to different ecosystem attributes (i.e. composition, structure, functioning) can help ensure that different modes of variability within the ecological system are represented and thus enable comprehensive assessment of the ecosystem state (Racault et al. 2014). In situ or remote-sensed measurements of chlorophyll concentration provide key information about the structure of phytoplankton populations. Using chlorophyll concentration and irradiance observations, algorithms can be implemented to estimate primary production (PP), providing a measure of the rate of conversion of inorganic carbon in CO_2_ to organic carbon by photosynthesis, which is key to assess ecosystem functioning. Also based on surface chlorophyll concentration, phenological algorithms can be applied to estimate the specific timings of important events in the phytoplankton growing period and provide further information on the ecosystem functioning.

Ocean-colour sensors on satellites can provide estimates of chlorophyll concentration at high spatial and temporal resolutions and at global scale. Because they provide data consistently and frequently and over long periods of time, they are suitable for computations of several ecological indicators (including PP and phenology) and for studying inter-annual variations and long-term trends in the state of the marine ecosystem. However, ocean-colour sensors do have a finite lifespan, and differences in instrument design and algorithms make it difficult to compare data from multiple sensors. When overlapping data are available from two or more sensors, such data can be used to establish inter-sensor bias and correct for it. The ESA Ocean Colour CCI (OC-CCI) has merged ocean-colour data using the Sea-viewing Wide Field-of-View Sensor (SeaWiFS 1997–2010), the Moderate-Resolution Imaging Spectroradiometer (MODIS 2002-present) and the MEdium-Resolution Imaging Spectrometer (MERIS 2002–2012) to provide the first 17-year (1997 to present) global scale, high-quality, bias-corrected and error-characterised data record of ocean colour (Sathyendranath et al. 2016). Furthermore, implementation of the coupled ocean–atmosphere POLYMER atmospheric correction algorithm (MERIS period) has increased significantly the coverage of chlorophyll observations (Steinmetz et al. 2011; Racault et al. 2015; Sathyendranath et al. 2016). The improvements realised in the OC-CCI products will help us to enhance evidence for, and improve confidence in, our understanding of the impacts of climate variability and change on the marine ecosystem.

Recent research has shown that variations in the abundance and phenology of phytoplankton populations can profoundly alter: (1) the efficiency of the biological pump, with inevitable impact of the global carbon cycle and (2) the interactions across trophic levels (Edwards and Richardson 2004), which can engender trophic mismatch with deleterious impact on the survival of commercially important fish and crustacean larvae (Platt et al. 2003; Koeller et al. 2009; Lo-Yat et al. 2011). The high turnover rate of phytoplankton, which is tightly coupled to environmental perturbations, makes them ideal indicators to study the first-level biological response to environmental changes. The main drivers of variations in phytoplankton populations include light and nutrient availability, which may be modulated by stratification, mixing, upwelling and riverine inputs. Perturbations in these physical processes may be characterised using observations of sea surface temperature (SST), net heat flux, winds, rainfall and sea surface height (SSH) and are broadly related to large-scale patterns of climate variability.

In the Earth’s climate system, the El Niño–Southern Oscillation (ENSO) is a dominant driving force of climate variability, involving warm (El Niño) and cold (La Niña) episodes with a typical periodicity of 2–7 years (McPhaden et al. 2006). The occurrence of ENSO episodes is characterised by anomalous changes in trade wind intensity and SST in the tropical Pacific. The planetary influence of ENSO is induced through a complex suite of ocean–atmosphere feedbacks, tropical–extratropical interactions and atmospheric teleconnections. The specific influence of each of these remote forcing mechanisms is not fully determined yet, but they can severely disrupt temperature and rainfall patterns, storm tracks and cyclone trajectories (Cai et al. 2015), with knock-on effects on crops, vector diseases (Martinez-Urtaza et al. 2016), and on marine ecosystem composition, structure and functioning (e.g., Behrenfeld et al. 2001; Jackson et al. 2011; Masotti et al. 2011; Racault et al. 2012).

This paper provides an overview of phytoplankton responses to the ENSO mode of climate variability based on a suite of ecological indicators (i.e. chlorophyll concentration, primary production, phenological metrics) estimated using ESA CCI ocean-colour observations in the global oceans (Sathyendranath et al. 2016). It explores and discusses the use of phenological metrics to identify occurrences of changes in environmental conditions and specifically of ENSO-related changes in environmental conditions. The environmental variables are based on ESA CCI-SST (Merchant et al. 2014) and CCI-SSH (Ablain et al. 2015) observations, and ECMWF ERA Interim reanalysis products of winds. Finally, the phenological responses to ENSO are used as a framework to help us understand further the mechanisms driving variability in phytoplankton populations.

## Phenology Based on Ocean-Colour Observations

**Fig. 1:**
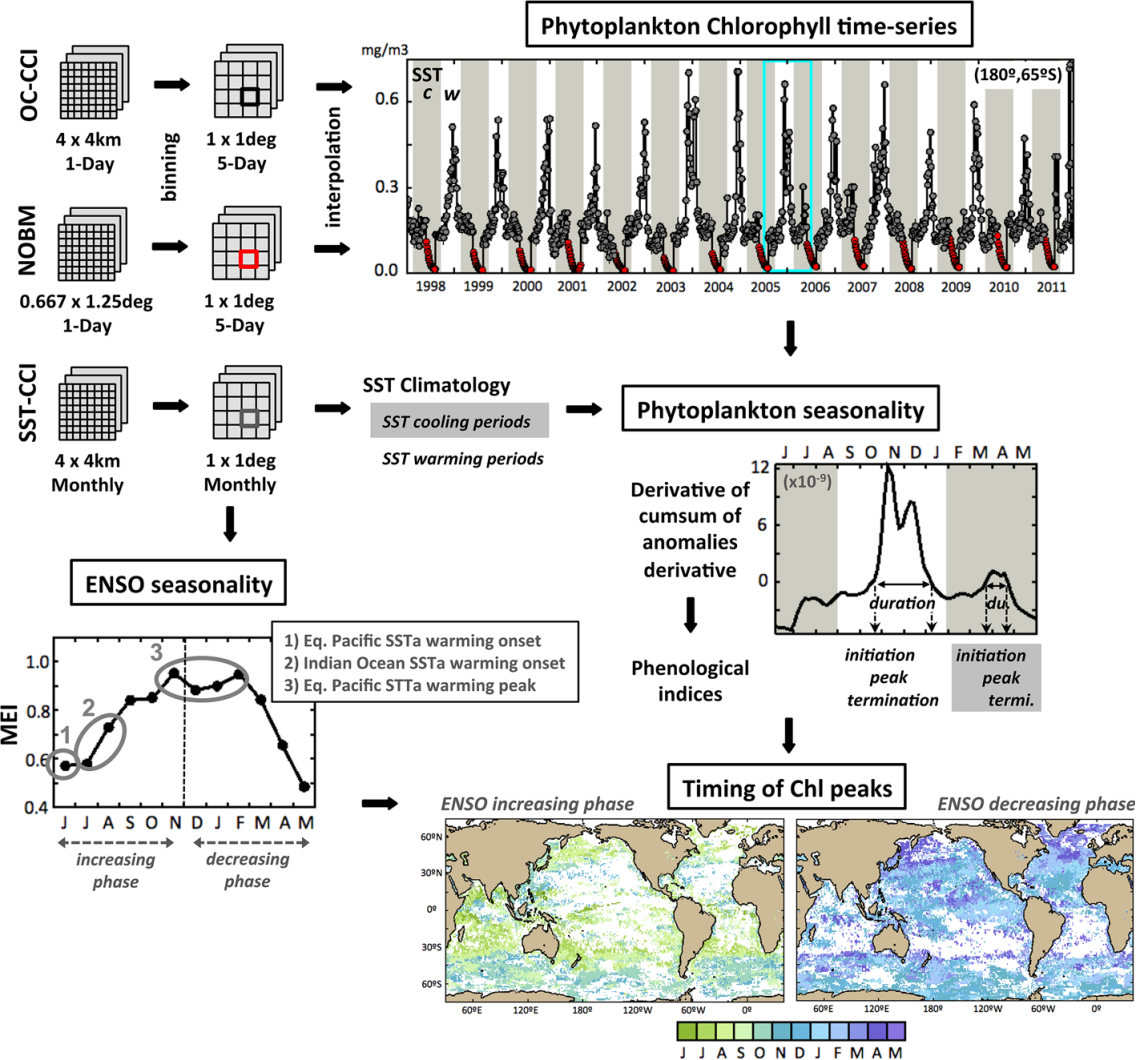
Schematic diagram of the phenology algorithm. The methodology to estimate two chlorophyll peaks per annual cycle has been based on Racault et al. (2015) and developed further. Ocean Colour Climate Change Initiative data were used over the period 1998–2009 at 5-day resolution. NOBM model data of chlorophyll concentration were estimated at 5-day resolution and used to fill persistent missing data due to high solar zenith angle at higher latitudes (*red dots* in the Chlorophyll time-series). Climatology of Sea Surface Temperature SST-CCI data were used to provide time boundaries to identify chlorophyll peaks in the annual cycle. Multivariate ENSO Index MEI was used to separate the annual cycle in two phases (increasing and decreasing ENSO phases). The plot of the derivative of the cumulative sum of chlorophyll anomalies and maps of timing of chlorophyll peak during ENSO increasing and decreasing phases are displayed here as an example, for the 12-month period between June 2005 to May 2006

Phenological metrics of timings of initiation, peak and termination, and duration of the phytoplankton growing period (Platt and Sathyendranath 1996; Platt and Sathyendranath 2008; Racault et al. 2012) can be calculated based on relative changes in the concentration of chlorophyll. In the present paper, the analysis uses Level-3 ESA OC-CCI chlorophyll dataset at 5-day temporal resolution and 1 × 1 degree spatial resolution over the period 1998–2009. The resolution has been chosen to minimise gaps in the data while retaining maximum resolution in time. A schematic representation of the phenological method is presented in Fig. 1. The phenological algorithm permits us to estimate up to two phytoplankton growing periods per year. It is based on a threshold criterion (i.e. chlorophyll long-term median plus 5 %) and calculation of the derivative of the cumulative sum of chlorophyll anomalies. The latter method had been initially developed at regional scale (i.e. the Red Sea) based on OC-CCI climatology at 8-day resolution (Racault et al. 2015). Here, the method has been further developed to be compatible with multi-annual chlorophyll time-series in the global oceans, and at an improved 5-day temporal resolution. Furthermore, construction of complete chlorophyll time-series (no data gaps) was achieved: (1) by applying linear interpolation to fill missing data due to cloud cover and (2) by inserting NASA Ocean Biogeochemical Model (NOBM, Gregg and Casey 2007; Gregg and Rousseaux 2014) chlorophyll data to fill persistent missing data due high solar zenith angle in winter at high latitudes. Such gap-free chlorophyll time-series are required to compute cumulative sums of anomalies and then estimate phenological indices. The integration of the NOBM data did not introduce bias in the time-series used to estimate the phenological analysis. This was apparent in the filled chlorophyll time-series and in the calculation of the cumulative sum (see panel “Phytoplankton time-series” in Fig. 1). In addition, to account for the large variability in timing of occurrence of the phytoplankton growing periods in the global oceans (Racault et al. 2012), SST seasonal cycle was used to define specific time intervals during which phenological indices were estimated: (1) during SST warming phase and (2) during SST cooling phases (white and grey shaded areas, respectively, in Fig. 1). This is a further improvement compared to the initial algorithm of Racault et al. (2015), which was based on fixed delineation of SST periods.

The phenological metrics and threshold criterion were estimated on a pixel-by-pixel basis. The timings of initiation and termination of phytoplankton growth are defined when the cumulative sum of chlorophyll changes sign (i.e. going from negative to positive and vice versa, respectively, Fig. 1). The sign changes correspond to the time when the chlorophyll concentrations rise above and fall below the relative threshold criterion (Siegel et al. 2002; Racault et al. 2012). Finally, the duration of the growing period is estimated as the time elapsed between initiation and termination. Given that phytoplankton response to ENSO has been demonstrated in the tropics, subtropics and during austral and boreal summer periods at high latitudes in the South and North Hemispheres, respectively (Behrenfeld et al. 2001, Yoder and Kennelly 2003, Messié and Chavez 2012), information about ENSO seasonal cycle was also taken into account in the development of the phenological algorithm. In particular, the time boundaries of the annual cycles were delineated over the period from June (of year *t*) to May (of year *t* + *1*) (i.e. spanning two calendar years). This 12-month delineation period was chosen to follow the seasonality of ENSO activity, generally peaking in the month of November to January (higher SST anomalies in the Equatorial Pacific, Fig. 1). The timings of chlorophyll peaks and phytoplankton growing periods were classified based on phases of increasing and decreasing ENSO anomaly.

In subpolar regions, nutrients are generally replenished during the winter season through enhanced mixing of the water column, and phytoplankton growth is primarily limited by light availability, which may be enhanced in spring when net heat flux becomes positive and the water-column stratifies. In these conditions, the timing of chlorophyll peak follows the latitudinal increase in light availability (Fig. 1) from the months of ~July to November in the Southern Hemisphere (i.e. ENSO increasing phase) and from the months of ~January to May in the Northern Hemisphere (i.e. ENSO decreasing phase). In the tropics and subtropics, light is plentiful all-year round, and phytoplankton growth is primarily limited by nutrient availability, which is enhanced by water-column mixing following environmental perturbations. In these regions, the timing of phytoplankton growth is not seasonal and may occur throughout the course of an annual cycle (Fig. 1).

The phenology indices presented in this paper were compared with, and shown to be consistent with, those in the literature, for instance results based on in situ or satellite observations at regional scale for North Atlantic (González Taboada and Anadón 2014; Cole et al. 2015), Southern Ocean (Thomalla et al. 2011; Carranza and Gille 2015) and North Pacific (Sasaoka et al. 2011), as well as previously published results for the global oceans (Racault et al. 2012). The increased resolution of the chlorophyll composites from 8 to 5 days was made possible by the significant improvement in data coverage in the OC-CCI product. Initially, the indices were estimated both at 5- and 8-day resolutions using the improved phenological algorithm, and the results from these two resolutions were seen to be consistent with each other. Hence, only the analysis at 5-day resolution is shown here.

**Fig. 2:**
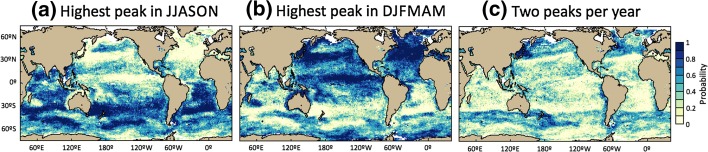
Probability of occurrence of chlorophyll peaks based on the phenological algorithm implemented using OC-CCI chlorophyll data at 5-day resolution. **a** Probability that the main chlorophyll peak occurs during the months of June to November JJASON (i.e. ENSO increasing phase, see Fig. 1); **b** Probability that the main chlorophyll peak occurs during the months of December to May DJFMAM (i.e. ENSO decreasing phase, see Fig. 1); and **c** Probability to have two chlorophyll peaks per year. If there are two chlorophyll peaks in 1 year, the main chlorophyll peak is defined by the peak with the higher amplitude (i.e. higher maximum chlorophyll value)

The present phenological algorithm further allows us to provide, in the global oceans, estimates of the probability that: (1) the main chlorophyll peak occurs during increasing or decreasing ENSO phase (Fig. 2a, b) and (2) two chlorophyll peaks occur each year (Fig. 2c). North and South Hemispheres seasonalities are apparent in this analysis: the main growing period (defined by the peak with the highest amplitude) is shown to occur during the months of June to November (i.e. JJASON in Fig. 2a) in the South Hemisphere, whereas it occurs during the months of December to May (i.e. DJFMAM in Fig. 2b) in the North Hemisphere. Interestingly, regions showing high probability to have a main chlorophyll peak in DJFMAM (i.e. predominantly found in the North Hemisphere) can also be found in the South Hemisphere, in the Pacific Ocean tropics and subtropics, off the east coast of Madagascar and in the Mozambique Channel, and along the west and northwest coast of Australia. The probability to have two chlorophyll peaks per year is almost zero in the tropics and subtropics, while it increases almost symmetrically towards higher latitudes in the North and South Hemispheres (Fig. 2c). Moreover, it is noteworthy that in high-latitude regions, the probability to have two chlorophyll peaks per year reaches values of ~0.5, indicating that two peaks in chlorophyll are only observed in ~half of the years during the period 1998–2009 (i.e. approximately half of the years present two peaks and the other half present one peak per year). The latter probability estimates, which are based on satellite observations, were compared and showed consistency with the latitudinal variations in the occurrence of phytoplankton blooms obtained in the North Atlantic using a model based on simple theoretical assumptions (Platt et al. 2009). This model demonstrates that the main driver explaining the variations in the probability of occurrence of two chlorophyll peaks per year is the latitudinal variations in the strength and periodicity of the initial forcing (i.e. variations in the magnitude in the total daily irradiance). Another approach based on 1000 a posteriori simulations from a model fitted to remote-sensed observations of chlorophyll concentration (15 consecutive seasonal cycles from 1998/1999 to 2012/2013) has permitted assessment of the probability of detecting different peaks in chlorophyll concentration and their timing in the Atlantic Ocean (between 15°S and 80°N; González Taboada and Anadón 2014). The authors showed higher probability of occurrence of two chlorophyll peaks per year in the North Atlantic subtropical region, which is consistent with the analysis presented in this paper.

The processes driving the inter-annual variability in phytoplankton phenology have been investigated at regional and global scales (e.g., Henson et al. 2009; Thomalla et al. 2011; Racault et al. 2012; Brody et al. 2013). Relationships have been demonstrated between the timing of phytoplankton growth and the timing of light availability and water-column stratification at high latitudes, and between the timing of phytoplankton growth and the timing of deepening of the mixed layer depth (as a proxy for water-column mixing and nutrient availability) in the tropics and subtropics. In the following sections, we focus on the influence of ENSO on phytoplankton phenological variability and review some of the possible driving processes available from satellite observations of SSH and SST.

## ENSO Impact on Chlorophyll, Primary Production and Phenology

ENSO activity, consisting of irregular El Niño and La Niña episodes, can profoundly impact marine ecosystem indicators. During the 1997–1999 El Niño/La Niña transition period, phytoplankton biomass increased by 10 % globally (Behrenfeld et al. 2001), and new production (dependent on new nitrogen) varied by more than a factor of two in the Equatorial Pacific (Turk et al. 2001). Short-term variability (less than one decade) in chlorophyll concentration, primary production and phenology of phytoplankton populations have been shown to correlate with ENSO variability in the Equatorial regions and in the global oceans albeit with marked regional differences (Yoder and Kennelly 2003; Behrenfeld et al. 2006; Vantrepotte and Mélin 2011; Chavez et al. 2011; Messié and Chavez 2012; Racault et al. 2012; Raitsos et al. 2015). Regional variability may weaken or enhance long-term trends, which may be further modulated by decadal oscillations in physical oceanographic conditions. In particular, the influence of ENSO and regional climate oscillations on SST and phytoplankton has been investigated in the North Atlantic (e.g., ENSO and North Atlantic Oscillation, Lee et al. 2008), in the North Pacific (e.g., ENSO and Pacific Decadal Oscillation, Di Lorenzo et al. 2008; Martinez et al. 2009), in the Indian Ocean (e.g., ENSO and IOD, Saji et al. 1999; Brewin et al. 2012; Currie et al. 2013), and in the Southern Ocean (e.g., ENSO and Southern Annular Mode, Soppa et al. 2016).

One of the most widely used environmental indices for assessing specifically the impact of El Niño on ocean biology is the Multivariate ENSO Index (MEI) (Wolter and Timlin 1993) (Table 1). The MEI encapsulates short-term variations of coupled ocean–atmosphere processes rooted in the tropical Pacific. Specifically, the MEI is defined as the first seasonally varying principal component of six atmosphere–ocean variable fields in the tropical Pacific basin (i.e. sea level pressure, zonal and meridional wind components, sea surface and air temperatures, and total cloudiness).

**Table 1:** ENSO characteristics during the period of study 1998–2009

	Years										
	98/99	99/00	00/01	01/02	02/03	03/04	04/05	05/06	06/07	07/08	08/09
MEI sign	–	–	–	+	+	+	+	–	+	–	–
Event type	LN	LN	LN		CP EN	CP EN		LN	CP EN	LN	LN

The El Niño and La Niña events are defined based on variations of the Multivariate ENSO Index by ±0.5 Standard Deviation. The classification of Central Pacific El Niño events is based on Yu et al. (2012) and Radenac et al. (2012)

*EN* El Niño, *CP* Central Pacific, *LN* La Niña

Linear regression analyses between MEI and annual mean chlorophyll concentration anomalies, between MEI and annual mean primary production anomalies, and between MEI and phenological metric anomalies can be used to characterise some of the variations in phytoplankton populations associated with ENSO. In this paper, linear regression analyses are performed based on annual mean of MEI values based on the Wolter and Timlin (1993) dataset, the ESA OC-CCI project chlorophyll dataset (Level 3, Mapped, 1 × 1 degree and monthly resolutions) (Sathyendranath et al. 2016), the Transboundary Waters Assessment Programme (GEF-TWAP) primary production dataset (Level 4, Mapped, 1 × 1 degree and monthly resolutions) and the phenological datasets presented in Sect. 2. The phenological indices can be estimated only when a complete seasonal cycle of chlorophyll is available. As the OC-CCI data record begins in September 1997 (with the SeaWiFS mission), the indices could not be estimated for the global oceans, during the 1997–1998 extreme El Niño event. However, the indices could be estimated during the following ENSO events over the period June 1998 to May 2009. Furthermore, the period of study (1998–2009) is marked by a major La Niña event in 1998/1999, a moderate La Niña event in 2007/2008 and a period of quasi-continuous positive MEI values between 2002 and 2007, which are characterised by anomalous SST warming in the central equatorial Pacific (Table 1). The meridional position (i.e. central or eastern Pacific) and amplitude of the SST anomalies may be classified as different extreme types of El Niño (Capotondi et al. 2015): the Eastern Pacific (EP El Niño), also referred to as the “typical” or canonical El Niño, characterised by anomalous SST warming in the eastern tropical Pacific; and the Central Pacific (CP El Niño), variously referred to as El Niño Modoki (Pseudo El Niño; Ashok et al. 2007; Kao and Yu 2009), warm-pool El Niño (Kug et al. 2009), or dateline El Niño (Larkin and Harrison 2005), and characterised by ocean warming anomalies occurring in the central tropical Pacific. The influence of these two extreme types of El Niño can lead to significantly different perturbations of environmental conditions and biological responses (e.g., Ashok and Yamagata 2009, Yu et al. 2012, Gierach et al. 2012, Radenac et al. 2012). Finally, the period of study from 1998 to 2009 was also chosen as it spans the availability of all of the different data records: OC-CCI chlorophyll (1997-present), TWAP primary production (1998–2010), phenology (1998–2009) and MEI (1950-present).

### ENSO Impact on Phytoplankton Phenology

**Fig. 3:**
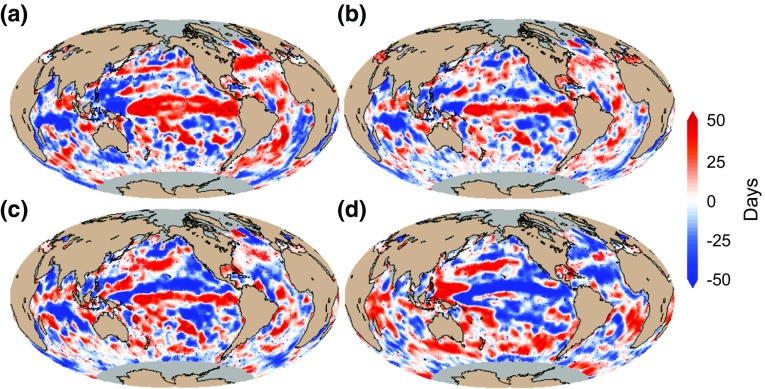
ENSO impact on phytoplankton phenology estimated using linear regression analysis between **a** MEI and anomalies of timing of initiation of phytoplankton growing period, **b** MEI and anomalies of timing of peak, **c** MEI and anomalies of timing of termination, and **d** MEI and anomalies of duration. Increase and decrease are indicated by positive (*red*) and negative (*blue*) anomalies, respectively. The phenological metrics were estimated over the period 1998–2009 based on OC-CCI chlorophyll data. In all panels, *red and blue stippling* indicates where the linear regression coefficients are significant at the 90 % confidence level

**Fig. 4:**
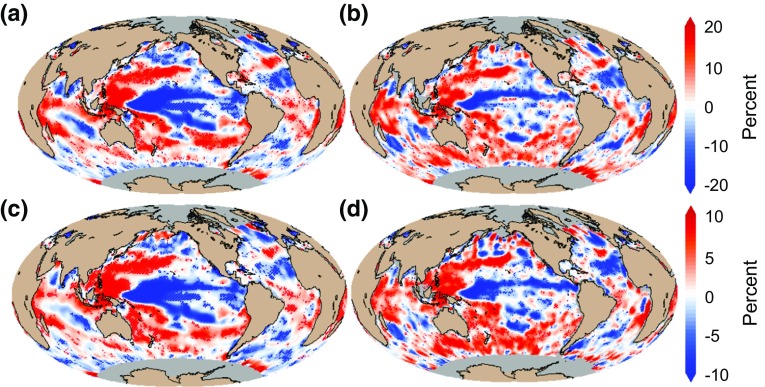
ENSO impact on annual and phenology-based chlorophyll concentration and primary production estimated using linear regression analysis between **a** MEI and annual mean anomalies of chlorophyll concentration, **b** MEI and mean anomalies of chlorophyll concentration over the duration of the phytoplankton growing period, **c** MEI and annual mean anomalies of primary production and **d** MEI and mean anomalies of primary production over the duration of the phytoplankton growing period. Increase and decrease are indicated by positive (*red*) and negative (*blue*) anomalies, respectively. Chlorophyll data are on OC-CCI data product, and primary production data are from TWAP (based on the algorithm of Platt and Sathyendranath (1988) were analysed during the period 1998–2009. In all panels, *red and blue stippling* indicates where the linear regression coefficients are significant at the 90 % confidence level

During positive phase of the MEI (Fig. 3a–c), the timings of initiation, peak and termination show delays of from ~25 to 40 days (positive anomalies) in tropical and extratropical regions of the central and eastern Pacific Ocean, in the subtropical regions of the Indian Ocean, in tropical and subtropical regions of the Atlantic Ocean, and also towards higher latitudes, between 40 and 50°N in the North Atlantic, and between 20 and 50°S in the western side of the South Atlantic. Conversely, the timings of the phytoplankton growing period are observed to occur earlier (between −15 and −30 days, negative anomalies) in the eastern equatorial region of the Pacific Ocean, in the equatorial region of the Indian Ocean and in large regions of the Southern Ocean and the eastern side of the South Atlantic Ocean. It is noteworthy that higher anomalies are observed in the timing of initiation compared with the timings of peak and termination. Furthermore, the regional changes observed in response to ENSO are coherent between the ecosystem indices: when initiation of the phytoplankton growing period is delayed, the timing of peak is also delayed and the timing of termination is advanced, leading to shorter duration and lower mean annual chlorophyll concentration and primary production (Figs. 3a, d, 4a, c).

### ENSO Impact on Chlorophyll and Primary Production

During positive phase of the MEI (Fig. 4a, c), annual mean chlorophyll concentration and primary production anomalies show marked decreases in tropical and extratropical regions of the central and eastern Pacific Ocean, in the subtropical and subpolar regions of the North Atlantic Ocean (between 10 and 20°N and polewards of 40°N) and in the subtropical region of the Indian Ocean. Conversely, increases in chlorophyll concentration and primary production are observed in the western Pacific Ocean, as well as over large regions of the Southern Ocean, and the equatorial region of the Indian Ocean. ENSO-related changes in chlorophyll concentration are larger, varying by ±20 % compared with the changes observed in primary production, varying by ±10 %. The changes are expressed in per cent rather than in absolute values because chlorophyll concentration can span three orders of magnitude and, hence, the values can be more readily interpreted and compared when expressed in relative terms.

To refine estimations of ENSO-related changes in phytoplankton, mean anomalies of chlorophyll concentration and primary production are also calculated specifically during the time interval between the initiation and termination of the main phytoplankton growing period each year. Globally, phytoplankton responses show similar pattern between the annual mean and phenology-based mean, indicating that the ENSO mode of variability dominates the observed annual variations in chlorophyll and primary production. Regionally, phenology-based responses present larger increases in chlorophyll and primary production in the Southern Ocean, and the Indian Ocean, and spatially more defined delineation of increases in the Atlantic Ocean. The decreases in phytoplankton concentration and production observed in the central and eastern tropical and subtropical Pacific Ocean are also more limited in extent (constrained to the equatorial Pacific region) when using the phenology-based estimates. The latter estimates further highlight larger decreases in the subtropical North Atlantic Ocean, and a marked decrease in phytoplankton of the southwest coast of Madagascar, which is quite prominent compared with the increases in chlorophyll and primary production observed in the surrounding waters. Regional and local differences between annual and phenology-based mean anomalies of chlorophyll and primary production are also observed in the Gulf of Guinea large marine ecosystem.

### Emergent Properties in Ocean-Colour Indices

**Fig. 5:**
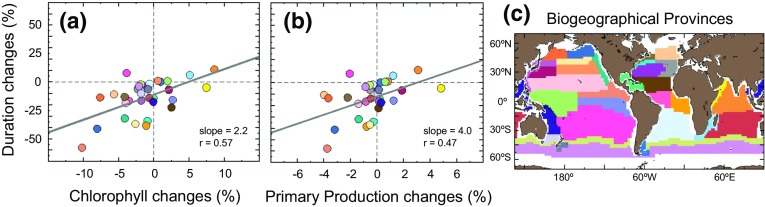
Emergent properties in ecological indicator responses to ENSO activity. **a** Relationship between relative changes in chlorophyll concentration and duration of phytoplankton growing period between years of positive ENSO and years of negative ENSO indices; **b** Relationship between relative changes in primary production and duration of phytoplankton growing period between years of positive ENSO and years of negative ENSO indices. The *colour of each dot* indicates the changes for each biogeochemical province (Longhurst 1998) delineated in panel **c**)

Relationships between ENSO-related responses shown in ecological indicators can be explored using linear regression analysis between relative changes in duration of phytoplankton growth and chlorophyll concentration, and between duration and primary production. Each ecological indicator is estimated using climatologies of positive and negative MEI years over the period 1998–2009. In the latter period, positive MEI years include 2001/2002 to 2004/2005, 2006/2007 and negative MEI years include 1998/1999 to 2000/2001, 2005/2006, 2007/2008, 2008/2009 (Table 1). The relative difference between responses to positive and negative MEI is computed for the annual mean chlorophyll concentration, annual mean primary production and the duration of phytoplankton growing period. The regression analysis is performed first on a pixel-by-pixel basis, and then the results are averaged in each biogeographical province (Fig. 5). This averaging procedure allows us to weight evenly the influence of ENSO in the tropics, subtropics and subpolar provinces. The partitioning of the provinces is based on physical, chemical and biological oceanographic knowledge and provides comprehensive geographical units supporting scientific findings interpretation and extrapolation (Longhurst 1998).

Based on the linear regression analyses, the relative changes observed in annual mean chlorophyll can explain 57 % of the relative changes in duration, and the relative changes observed in annual mean primary production can explain 47 % of the relative changes in duration (Fig. 5a, b, respectively, *p* < 0.01). The sign and magnitude of the slopes are positive and greater than one, such that increases in MEI-associated changes in duration are accompanied by a twofold increase in the response of chlorophyll to MEI, and increases observed in MEI-associated changes in duration are accompanied by a fourfold increase in the response of primary production to MEI. Largest MEI-related increases in chlorophyll, primary production and duration are observed in the Indian Ocean Monsoon Gyre province and in the Agulhas and Somali Current Large Marine Ecosystems province, whereas the largest decreases in the indicator values are observed in the eastern Equatorial Pacific, subtropical and subpolar North Pacific regions (Fig. 5a, b). The emergence of linear relationships amongst ENSO-responses of indicators, which are initially measured in different units (i.e. mgChl m^−3^ for chlorophyll concentration, mgC m^−2^ year^−1^ for primary production, and days for duration), can be particularly useful to analyse and compare indicators estimated from non-continuous data records, and when inter-sensor bias correction cannot be quantified (for instance, to compare changes in phytoplankton population between non-overlapping ocean-colour sensors CZCS (1978–1986) and contemporary sensors, starting in 1997 with SeaWiFS, and follow-on sensors in 2002 with MODIS or MERIS).

## Phenology-Based Biophysical Responses to ENSO

**Fig. 6:**
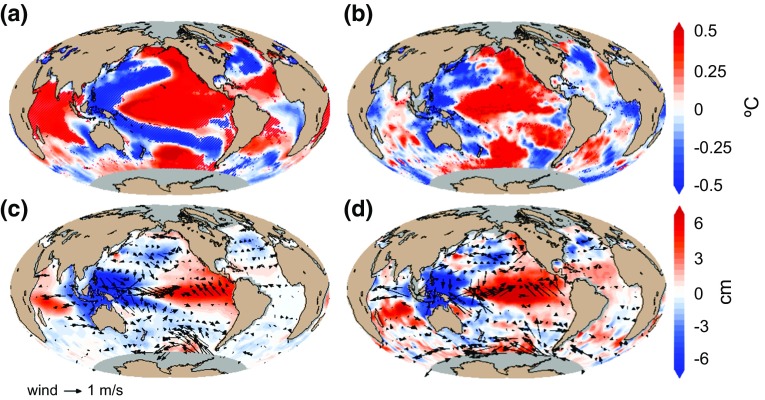
ENSO influence on annual and phenological mean physical conditions estimated using linear regression analysis between **a** MEI and annual mean anomalies of SST, **b** MEI and mean anomalies of SST over the duration of the phytoplankton growing period, **c** MEI and annual mean anomalies of SSH and mean wind vectors and **d** MEI and mean anomalies of SSH and mean wind vectors over the duration of the phytoplankton growing period. SST and SSH datasets are from ESA CCI program, and wind data are from ECMWF ERA Interim reanalysis. Increase and decrease are indicated by positive (*red*) and negative (*blue*) anomalies, respectively. In all panels, *red and blue stippling* indicates where the linear regression coefficients are significant at the 90 % confidence level over the entire period 1998–2009

Mechanistic understanding of the biological responses to ENSO activity is difficult to assess because they result from complex biophysical interactions. In this section, similarly to the analysis carried out with the ocean-colour data products, the influence of ENSO on oceanic physical variables are investigated using linear regression analyses between MEI and annual mean SST anomalies, between MEI and annual mean wind anomalies and between MEI and annual mean SSH anomalies (Fig. 6). Since sea level reflects on the integral effect of surface and subsurface processes (e.g., ocean warming or cooling), SSH anomaly can be used as a good indicator for upwelling anomaly for very large upwelling regions that extend a long way offshore, and large-scale open-ocean upwelling such as in the Equatorial Pacific (Fu and Cazenave 2001). In addition, as variations in phytoplankton populations are tightly coupled to changes in environmental conditions, phytoplankton responses can be used as sentinels, or indicators of other, less obvious, changes occurring in the environment. In this context, phenological metrics are used to estimate ENSO-related changes in physical conditions that occur specifically during the phytoplankton growing period. For this approach, anomalies in the physical variables are averaged over the duration of the main growing period (characterised by the highest chlorophyll peak in the year, Fig. 2), so as to provide refined (more targeted) detection of the period and magnitude of physical changes occurring in the environment.

The influence of ENSO estimated using annual mean and phenology-related mean physical variables present consistent patterns in the Pacific and Atlantic Oceans and show marked differences in the Indian Ocean (Fig. 6). During positive MEI phases, large decrease is observed in the west Pacific Ocean, forming a V-shape rooted in the Equatorial region, and extending to the subtropics and towards higher latitudes in subpolar regions. In contrast, the central and eastern equatorial Pacific Ocean present large increases in SST and SSH. The latter patterns are coherent with the observed influence of Central Pacific El Niño events (Ashok and Yamagata 2009, Gierach et al. 2012; Table 1), showing enhanced easterly trade winds in the east and westerlies in the west, which push warmer, nutrient-poor waters to the central-western Equatorial Pacific (Fig. 6c, d), and cause a deepening of the thermocline, enhanced stratification, a subsequent decrease in chlorophyll and primary production (Fig. 4), and a delay and shortening of the timing of initiation and duration of the phytoplankton growing period, respectively (Fig. 3). It is further noteworthy that the phytoplankton response is not limited to this “wind convergence” region, but rather extends across the equatorial Pacific from the central-western basin to the eastern basin. This is because the westerly wind anomalies in the western-central Pacific cause equatorial Ekman convergence, increasing sea level (Fig. 6c, d) and deepening the thermocline (Palanisamy et al. 2015). These signals propagate eastward as equatorial Kelvin waves, overcoming the effects of upwelling favourable easterly wind anomalies in the eastern basin. The increased sea level and deepened thermocline signals weaken the mean upwelling in these regions, which limit the transport of nutrients to the surface mixed layer, and thus reduce the chlorophyll concentration and primary production.

In the tropical regions of the Atlantic Ocean, during positive phases of the MEI, patterns of increasing SST and SSH and enhanced Equatorial easterlies are observed in the east (Fig. 6a, c), bringing nutrient-poor waters to around 15°N (Lübbecke and McPhaden 2012), and causing a decrease in chlorophyll and primary production (Fig. 4), as well as a delay and shortening of the timing of initiation and duration of phytoplankton growth (Fig. 3). Although similar biological responses are observed towards higher latitudes in the North Atlantic (Figs. 3, 4), the underlying mechanism driving these responses is different: in higher latitudes, phytoplankton growth may be reduced and delayed, when water-column mixing is too high and light availability too low. During positive phases of the MEI over the period of study, enhanced cyclonic wind is observed in the North Atlantic (Fig. 6d), enhancing water divergence, which brings cooler-deeper water to the surface and decreasing SST and SSH (Fig. 6). In these conditions, water-column stratification is reduced, which subsequently delays phytoplankton growth, and decreases chlorophyll, primary production and duration of the growing period (Figs. 3, 4).

In the Indian Ocean, the ENSO-related changes estimated using annual means show increases in SST and SSH across the western and eastern basin (Fig. 6a, c), whereas based on the phenological mean, a weakly significant dipole pattern of decreasing SST in the west and increasing SST in the east is observed, together with a band of decreasing SSH along the equator (Fig. 6b, d). In the wind forcing responses, based on annual mean anomalies, the south tropical Indian Ocean is dominated by easterlies (Fig. 6c), whereas based on phenological mean anomalies, the forcing is characterised by strengthened easterly trade winds blowing both above and below the Equator (Fig. 6d). Over the south tropical Indian Ocean, increased SSH and SST appear in the western and central basin during positive ENSO phase (Fig. 6). This region is the mean upwelling zone of the Indian Ocean that is characterised by the thermocline ridge (i.e. TRIO region; Trenary and Han 2012). The high sea level (deepened thermocline) is primarily forced by easterly wind (Fig. 6c, d), which reduces the mean upwelling and thus reduce chlorophyll and primary production (Fig. 4a, c). In this basin, the phenology-based analysis appears to reveal coherent changes in wind forcing and responses of phytoplankton populations, which would have been more difficult to interpret from the analysis based on annual mean observations. Thus, phenological analysis can be suggested as a useful approach to help identify physical processes driving variations in phytoplankton populations in oceanic regions characterised by complex climate forcing (such as the monsoon in the Indian Ocean).

## Conclusions

Phytoplankton responses to ENSO, noticeable in the global oceans but with marked regional differences, are characterised by variations in timing of growing period between ±30 days, chlorophyll concentration between ±20 % and primary production between ±10 %. Such variations may have profound impact on the carbon cycle (i.e. changes in export production) and the functioning of the marine ecosystem (i.e. trophic match/mismatch). The patterns of variations in duration of phytoplankton growing period are shown to co-vary with variations in chlorophyll concentration and with variations in primary production. These emergent properties between ocean-colour metrics based on relative patterns (i.e. duration) and absolute changes in concentration and production rates (i.e. chlorophyll and primary production) may provide a relevant and alternative approach to support comparisons of ocean-colour products estimated from different sensors for which inter-calibration and bias correction processes cannot be carried out.

The analysis of phenology-based estimates of SST, SSH and wind data is shown to help refine the evaluation and understanding of the mechanisms of impact of ENSO on phytoplankton inter-annual variability at global and regional scales. The present overview highlights that in oceanic regions where ENSO may influence a climate system tied to annual variations in the solar cycle, such as in high latitudes, annual means of physical variables may be useful metrics to understand the mechanisms driving the regional biological variability. However, in oceanic regions where ENSO influences a climate system characterised by a seasonal reversal of the wind forcing, such as the monsoon in the Indian Ocean, which can drive phytoplankton responses that are equal in strength but opposite in direction, then the estimation of phenology-based mean of physical variables may be necessary to evaluate the mechanisms driving the biological responses and provide a more comprehensive understanding of the integrated processes. Thus, phenological studies on ENSO impact have broader implications for climate research: above and beyond understanding biological responses to shorter-term climate variability, they may be used to help us improve predictions on the impact of climate change on the marine ecosystem.

## References

[CR1] Ablain M, Cazenave A, Larnicol G, Balmaseda M, Cipollini P, Faugère Y, Fernandes MJ, Henry O, Johannessen JA, Knudsen P, Andersen O, Legeais J, Meyssignac B, Picot N, Roca M, Rudenko S, Scharffenberg MG, Stammer D, Timms G, Benveniste J (2015). Improved sea level record over the satellite altimetry era (1993–2010) from the climate change initiative project. Ocean Sci.

[CR2] Ashok K, Yamagata T (2009). The El Niño with a difference. Nature.

[CR3] Ashok K, Behera SK, Rao SA, Weng H, Yamagata T (2007). El Niño Modoki and its possible teleconnection. J Geophys Res.

[CR4] Behrenfeld MJ, Randerson JT, McClain CR, Feldman GC, Los SO, Tucker CJ, Falkowski PG, Field CB, Frouin R, Esaias WE, Kolber DD, Pollack NH (2001). Biospheric primary production during an ENSO transition. Science.

[CR5] Behrenfeld MJ, O’Malley RT, Siegel DA, McClain CR, Sarmiento JL, Feldman GC, Milligan AJ, Falkowski PG, Letelier RM, Boss MS (2006). Climate-driven trends in contemporary ocean productivity. Nature.

[CR6] Brewin RJW, Hirata T, Hardman-Mountford NJ, Lavender SJ, Sathyendranatha S, Barlow R (2012). The influence of the Indian ocean dipole on inter annual variations in phytoplankton size structure as revealed by earth observation. Deep Sea Res Part II.

[CR7] Brody SR, Lozier MS, Dunne JP (2013). A comparison of methods to determine phytoplankton bloom initiation. J Geophys Res.

[CR8] Cai W, Santoso A, Wang G, Yeh S, An S, Cobb KM, Collins M, Guilyardi E, Jin F, Kug J, Lengaigne M, McPhaden MJ, Takahashi K, Timmermann A, Vecchi G, Watanabe M, Wu L (2015). ENSO and greenhouse warming. Nat Clim Change.

[CR9] Capotondi (2015). Understanding ENSO diversity. Am Meteorol Soc.

[CR10] Carranza MM, Gille ST (2015). Southern Ocean wind-driven entrainment enhances satellite chlorophyll-a through the summer. J Geophys Res: Oceans.

[CR11] Chavez FP, Messié M, Pennington JT (2011). Marine primary production in relation to climate variability and change. Ann Rev Mar Sci.

[CR12] Cole HS, Henson S, Martin AP, Yool A (2015). Basin-wide mechanisms for spring bloom initiation: how typical is the North Atlantic?. ICES J Mar Sci.

[CR13] Currie JC, Lengaigne M, Vialard J, Kaplan DM, Aumont O, Naqvi SWA, Maury O (2013). Indian Ocean dipole and El Niño/southern oscillation impacts on regional chlorophyll anomalies in the Indian Ocean. Biogeosciences.

[CR14] Di Lorenzo E, Schneider N, Cobb KM, Franks PJS, Chhak K, Miller AJ, McWilliams JC, Bograd SJ, Arango H, Curchitser E, Powell TM, Rivière P (2008). North Pacific Gyre Oscillation links ocean climate and ecosystem change. Geophys Res Lett.

[CR15] Edwards M, Richardson AJ (2004). Impact of climate change on marine pelagic phenology and trophic mismatch. Nature.

[CR16] Fu L-L, Cazenave A (2001). Satellite altimetry and earth sciences: a handbook of techniques and applications.

[CR17] Gierach MM, Lee T, Turk D, McPhaden MJ (2012). Biological response to the 1997–98 and 2009–10 El Niño events in the equatorial Pacific Ocean. Geophys Res Lett.

[CR18] González Taboada F, Anadón R (2014). Seasonality of North Atlantic phytoplankton from space: impact of environmental forcing on a changing phenology (1998–2012). Glob Change Biol.

[CR19] Gregg WW, Casey NW (2007). Sampling biases in MODIS and SeaWiFS ocean chlorophyll data. Remote Sens Environ.

[CR20] Gregg WW, Rousseaux CS (2014). Decadal trends in global pelagic ocean chlorophyll: a new assessment integrating multiple satellites, in situ data, and models. J Geophys Res.

[CR21] Henson SA, Dunne JP, Sarmiento JL (2009). Decadal variability in North Atlantic phytoplankton blooms. J Geophys Res.

[CR22] Jackson T, Bouman HA, Sathyendranath S, Devred E (2011). Regional-scale changes in diatom distribution in the Humboldt upwelling system as revealed by remote sensing: implications for fisheries. ICES J Mar Sci.

[CR60] Kao H-Y, Yu J-Y (2009). Contrasting Eastern-Pacific and Central-Pacific types of ENSO. J Clim.

[CR23] Koeller P, Fuentes-Yaco C, Platt T, Sathyendranath S, Richards A, Ouellet P, Orr D, Skúladóttir U, Wieland K, Savard L, Aschan M (2009). Basin-scale coherence in phenology of shrimps and phytoplankton in the North Atlantic Ocean. Science.

[CR24] Kug J-S, Jin F-F, An S-I (2009). Two types of El Niño events: cold tongue El Niño and warm pool El Niño. Am Meteorol Soc.

[CR25] Larkin NK, Harrison DE (2005). On the definition of El Niño and associated seasonal average US weather anomalies. Geophys Res Lett.

[CR27] Lee SK, Enfield DB, Wang C (2008). Why do some El Niños have no impact on tropical North Atlantic SST?. Geophys Res Lett.

[CR28] Longhurst A (1998). Ecological geography of the sea.

[CR29] Lo-Yat A, Simpson SD, Meekan M, Lecchini D, Martinez E, Glazin R (2011). Extreme climatic events reduce ocean productivity and larval supply in a tropical reef ecosystem. Glob Change Biol.

[CR30] Lübbecke JF, McPhaden MJ (2012). On the inconsistent relationship between Pacific and Atlantic Niños. J Clim.

[CR31] Martinez E, Antoine D, D’Ortenzio F, Gentili B (2009). Climate-driven basin-scale decadal oscillations of oceanic phytoplankton. Science.

[CR32] Martinez-Urtaza J, Trinanes J, Gonzalez-Escalona N, Baker-Austin C (2016). Is El Niño a long-distance corridor for waterborne disease?. Nat Microbiol.

[CR33] Masotti I, Moulin C, Alvain S, Bopp L, Antoine D (2011). Large scale shifts in phytoplankton groups in the Equatorial Pacific during ENSO cycles. Biogeosciences.

[CR34] McPhaden MJ, Zebiak SE, Glantz MH (2006). ENSO as an integrating concept in earth science. Science.

[CR35] Merchant CJ, Embury O, Roberts-Jones J, Fiedler E, Bulgin CE, Corlett GK, Good S, McLaren A, Rayner N, Morak-Bozzo S, Donlon C (2014). Sea surface temperature datasets for climate applications from Phase 1 of the European Space Agency Climate Change Initiative (SST CCI). Geosci Data J.

[CR36] Messié M, Chavez FP (2012). A global analysis of ENSO synchrony: the oceans’ biological response to physical forcing. J Geophys Res.

[CR37] Palanisamy H, Cazenave A, Delcroix T, Meyssignac B (2015). Spatial trend patterns in the Pacific Ocean sea level during the altimetry era: the contribution of thermocline depth change and internal climate variability. Ocean Dyn.

[CR38] Platt T, Sathyendranath S (1988). Oceanic primary production: estimation by remote sensing at local and regional scales. Science.

[CR39] Platt T, Sathyendranath S (1996). Modelling primary production?. Aquabiology.

[CR40] Platt T, Sathyendranath S (2008). Ecological indicators for the pelagic zone of the ocean from remote sensing. Remote Sens Environ.

[CR41] Platt T, Fuentes-Yaco C, Frank K (2003). Spring algal bloom and larval fish survival. Nature.

[CR42] Platt T, White III, Zhai L, Sathyendranath S, Roy S (2009). The phenology of phytoplankton blooms: ecosystem indicators from remote sensing. Ecol Model.

[CR59] Racault M-F, Quéré C, Buitenhuis E, Sathyendranath S, Platt T (2012). Phytoplankton phenology in the global ocean. Ecol Indic.

[CR43] Racault MF, Platt T, Sathyendranath S, Ağirba E, Martinez Vicente V, Brewin R (2014). Plankton indicators and ocean observing systems: support to the marine ecosystem state assessment. J Plankton Res.

[CR44] Racault MF, Raitsos DE, Berumen ML, Brewin RJW, Platt T, Sathyendranath S, Hoteit I (2015). Phytoplankton phenology indices in coral reef ecosystems: application to ocean-color observations in the Red Sea. Remote Sens Environ.

[CR45] Radenac MH, Léger F, Singh A, Delcroix T (2012). Sea surface chlorophyll signature in the tropical Pacific during eastern and central Pacific ENSO events. J Geophys Res.

[CR46] Raitsos DE, Yi X, Platt T, Racault MF, Brewin RJW, Pradhan Y, Papadopoulos PV, Sathyendranath S, Hoteit I (2015). Monsoon oscillations regulate fertility of the Red Sea. Geophys Res Lett.

[CR47] Saji NH, Goswami BN, Vinayachandran PN, Yamagata T (1999). A dipole mode in the tropical Indian Ocean. Nature.

[CR48] Sasaoka K, Chiba S, Saino T (2011). Climatic forcing and phytoplankton phenology over the subarctic North Pacific from 1998 to 2006, as observed from ocean color data. Geophys Res Lett.

[CR49] Sathyendranath S, Brewin RJW, Brockmann C, Brotas V, Ciavatta S, Chuprin A, Couto AB, Doerffer R, Dowell M, Grant M, Groom S, Horseman A, Jackson T, Krasemann H, Lavender S, Martinez Vicente V, Mélin Moore TS, Müller D, Regner P, Roy S, Steinmetz F, Swinton J, Taberner M, Thompson A, Valente A, Zühlke M, Brando VE, Feldman G, Franz B, Frouin R, Gould Jr RW, Hooker S, Kahru M, Mitchell MG, Muller-Karger F, Sosik HM, Voss KJ, Werdell J, Platt T (2016) Creating an ocean-colour time series for use in climate studies: the experience of the ocean-colour climate change initiative. (Manuscript)

[CR61] Siegel DA, Doney SC, Yoder JA (2002). The North Atlantic spring phytoplankton bloom and Sverdrup's critical depth hypothesis. Science.

[CR50] Soppa MA, Volker C, Astrid B (2016). Diatom phenology in the Southern Ocean: mean patterns, trends and the role of climate oscillations. Remote Sens.

[CR51] Steinmetz F, Deschamps PY, Ramon D (2011). Atmospheric correction in presence of sun glint: application to MERIS. Opt Expr.

[CR52] Thomalla SJ, Fauchereau N, Swart S, Monteiro PMS (2011). Regional scale characteristics of the seasonal cycle of chlorophyll in the Southern Ocean. Biogeosciences.

[CR53] Trenary LL, Han W (2012). Intraseasonal-to-interannual variability of south indian ocean sea level and thermocline: remote versus local forcing. J Phys Oceanogr.

[CR54] Turk D, McPhaden MJ, Busalacchi AJ, Lewis M (2001). Remotely sensed biological production in the equatorial Pacific. Science.

[CR55] Vantrepotte V, Mélin F (2011). Inter-annual variations in the SeaWiFS global chlorophyll a concentration (1997–2007). Deep Sea Res Part I Oceanogr Res Pap.

[CR56] Wolter K, Timlin MS (1993) Monitoring ENSO in COADS with a seasonally adjusted principal component index. In: Proceedings of 17th climate diagnostics workshop (Norman, Oklahoma) 52–57 (NOAA/N MC/CAC, NSSL, Oklahoma Climate Survey, CIMMS and the School of Meteorology, Univ. Oklahoma, 1993)

[CR57] Yoder J, Kennelly M (2003). Seasonal and ENSO variability in global ocean phytoplankton chlorophyll derived from 4 years of SeaWiFS measurements. Glob Biogeochem Cycles.

[CR58] Yu JY, Zou Y, Kim ST, Lee T (2012). The changing impact of El Niño on US winter temperatures. Geophys Res Lett.

